# Forme rare de malformation anorectale avec une fistule recto urethrobulbaire: à propos d’un cas

**DOI:** 10.11604/pamj.2017.28.255.14299

**Published:** 2017-11-22

**Authors:** Othmane Alaoui, Hicham Abdellaoui

**Affiliations:** 1 Service de chirurgie pédiatrique viscérale, CHU Hassan II, Université Sidi Mohamed Ben Abdallah, Fès, Maroc.

**Keywords:** Malformation, fistule, imperforation, Malformation, fistula, imperforate

## Abstract

We here report the case of a male newborn admitted immediately after birth with imperforate anus. Physical examination of the anal margin showed imperforate anus; the examination of the external genitalia objectified scrotal bifidity with a fistula filled with meconium at the level of the penis root. During urinary catheterization the catheter passed through the fistula (A), suggesting a rare anorectal malformation with recto-uretrobulbar fistula. Malformation assessment was without abnormalities. The newborn was admitted to the operating room and clouding was performed during surgery by catheterization of the fistula using two 6 CH (1.98mm) Foley catheters, one passing through the rectum and the other passing through the bladder; a third foley catheter passed through the urethral meatus, objectifying the communication among the three catheters at the level of the recto-uretrobulbar fistula (B). The diagnosis of rare intermediate anorectal malformation was retained and colostomy was performed. The newborn underwent treatment based on perineal anorectoplasty with fistula closure at the age of 3 months. Anal dilatation was performed for 6 months. Colostomy closure was performed at the age of 9 months. Patient's evolution was favorable at 2-year follow-up.

## Image en médecine

We here report the case of a male newborn admitted immediately after birth with imperforate anus. Physical examination of the anal margin showed imperforate anus; the examination of the external genitalia objectified scrotal bifidity with a fistula filled with meconium at the level of the penis root. During urinary catheterization the catheter passed through the fistula (A), suggesting a rare anorectal malformation with recto-uretrobulbar fistula. Malformation assessment was without abnormalities. The newborn was admitted to the operating room and clouding was performed during surgery by catheterization of the fistula using two 6 CH (1.98mm) Foley catheters, one passing through the rectum and the other passing through the bladder; a third foley catheter passed through the urethral meatus, objectifying the communication among the three catheters at the level of the recto-uretrobulbar fistula (B). The diagnosis of rare intermediate anorectal malformation was retained and colostomy was performed. The newborn underwent treatment based on perineal anorectoplasty with fistula closure at the age of 3 months. Anal dilatation was performed for 6 months. Colostomy closure was performed at the age of 9 months. Patient's evolution was favorable at 2-year follow-up.

Nous rapportons le cas d'un nouveau né de sexe masculin admis à la naissance pour une imperforation anale chez qui l'examen physique a trouvé à l'examen de la marge anale une imperforation anale; l'examen des organes génitaux externes a objectivé une bifidité scrotale avec une fistule au niveau de la racine de la verge ramenant du méconium. Le sondage urinaire a noté l'issue de la sonde à travers la fistule (A) faisant suspecter une forme rare de malformation anorectale avec fistule recto urethrobulbaire. Un bilan malformatif a été réalisé revenant sans particularités. Le nouveau né a été admis au bloc opératoire et une opacification a été réalisé en per opératoire par un cathétérisme de la fistule par deux sondes de Foley charnière 6 l'une passant à travers le rectum et l'autre passant à travers la vessie; une troisième sonde de Foley a été mise par le méat urétral et objectivant la communication des trois sondes au niveau de la fistule dite recto urethro bulbaire (B). Le diagnostic a été retenu pour cette forme particulière de malformation anorectale intermédiaire et une colostomie a été réalisée. Le nouveau a bénéficié d'une cure de sa malformation ano rectale à l'âge de 3 mois ayant bénéficié d'une anorectoplastie avec fermeture de la fistule par voie périnéale. Des séances de dilatations anales ont été réalisées pendant 6 mois. La fermeture de la colostomie a été faite à l'âge de 9 mois. L'évolution a été favorable avec un recul de 2 ans.

**Figure 1:**
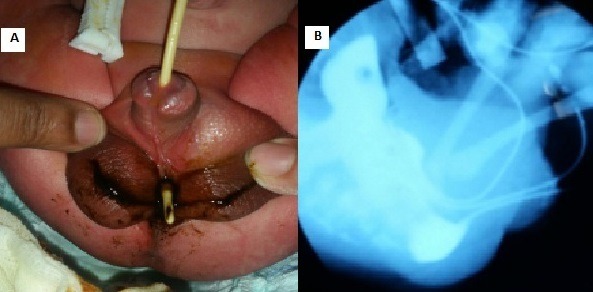
A) image clinique montrant l’issue de la sonde urinaire par la fistule; B) communication des trois sondes au niveau de la fistule recto urethro bulbaire

